# Clinical Characteristics and Prognosis of Incidentally Detected Lung Cancers

**DOI:** 10.1155/2015/287604

**Published:** 2015-02-03

**Authors:** S. Quadrelli, G. Lyons, H. Colt, D. Chimondeguy, A. Buero

**Affiliations:** ^1^Thoracic Oncology Centre, Buenos Aires British Hospital, Perdriel 74, C1280AEB Buenos Aires, Argentina; ^2^University of California, Irvine, CA 92697, USA

## Abstract

*Objective*. To evaluate clinical characteristics and outcomes in incidentally detected lung cancer and in symptomatic lung cancer.* Material and Methods*. We designed a retrospective study including all patients undergoing pulmonary resection with a curative intention for NSCLC. They were classified into two groups according to the presence or absence of cancer-related symptoms at diagnosis in asymptomatic (ASX)—incidental diagnosis—or symptomatic.* Results*. Of the 593 patients, 320 (53.9%) were ASX. In 71.8% of these, diagnosis was made by chest X-ray. Patients in the ASX group were older (*P* = 0.007), had a higher prevalence of previous malignancy (*P* = 0.002), presented as a solitary nodule more frequently (*P* < 0.001), and were more likely to have earlier-stage disease and smaller cancers (*P* = 0.0001). A higher prevalence of incidental detection was observed in the last ten years (*P* = 0.008). Overall 5-year survival was higher for ASX (*P* = 0.001). Median survival times in pathological stages IIIB-IV were not significantly different.* Conclusion*. Incidental finding of NSCLC is not uncommon even among nonsmokers. It occurred frequently in smokers and in those with history of previous malignancy. Mortality of incidental diagnosis group was lower, but the better survival was related to the greater number of patients with earlier-stage disease.

## 1. Introduction

Lung cancer is the most common cause of cancer mortality in the western world, accounting for approximately 5% of all deaths in many countries [1]. Currently less than 25–30% of patients present with localised, potentially curable disease. Five-year survival for those with pathological stage IA non-small cell lung cancer (NSCLC) is 73% whereas metastatic disease has a dismal prognosis (13% 5-year survival) [2].

Results from several studies suggest that frequent chest radiographic screening does not result in reduced lung cancer mortality, a conclusion reinforced by the Prostate, Lung, Colorectal, and Ovarian (PLCO) Cancer Screening Trial [3, 4].* In fact, some* studies suggest that frequent chest radiographic screening is associated with an 11% relative increase in lung cancer mortality compared with less frequent screening [3].

Randomized trials of screening low-dose computed tomography (LDCT) scans demonstrate that computed tomography (CT) is far more sensitive than chest radiography. CT is now considered the most suitable imaging screening modality. The National Lung Screening Trial (NLST) showed that, in heavy (30 pack-years or more) current or former (within 15 years) smokers between the ages of 55 and 75, three annual LDCT screens reduced lung cancer-specific mortality from 309 to 247 deaths per 100,000 person-years [5].

There are still many unanswered questions about the benefits and harms of those programs that could determine the ultimate success of the mass screening implementation. Additionally, despite expert guidelines for screening high-risk populations, most national health service providers have not implemented (and probably will not implement in the near future) mass lung cancer screening programs. One of the main concerns is that the extrapolation of findings from tightly controlled trials to real-life mass screening programs requires uniform standards and high quality controls not easily achievable in most institutions [6]. Consequently, the current practice is that the patients themselves or their physicians may choose early lung cancer detection on an individual basis. There is little information, however, on the clinical characteristics and outcomes of patients with incidentally detected early stage lung cancer from strictly controlled randomized trials.

The objective of this study was to analyze the clinical records of lung cancer patients who underwent surgical resection to evaluate the clinical characteristics and outcomes of patients with incidentally detected lung cancer and patients with symptomatic lung cancer.

## 2. Material and Methods

All patients undergoing pulmonary resection with a curative intention for non-small cell lung cancer (NSCLC) in the British Hospital in Buenos Aires between January 1986 and July 2009 were eligible for inclusion in this retrospective study. Our Thoracic Oncology Centre keeps a database of all patients evaluated, with data entered prospectively at the time of their initial evaluation. Patients were excluded if they had exhibited small cell lung cancer or a rare histological result.

Preoperative data included methods of diagnosis and a symptoms questionnaire, tobacco exposure history, and medical history. Only patients with complete and accurate preoperative data about indications for imaging were eligible. Patients were included only after institutional review board approval.

Preoperative staging was performed according to the 7th TNM classification system of the International Association for the Study of Lung Cancer [2] using chest computed tomography (CT) and abdominal CT or ultrasonography in all patients. Brain computed tomography or magnetic resonance imaging was done only in case of clinical suspicion of brain metastases. In cases of uncertain clinical or radiologic findings, further examinations were performed to exclude extrapulmonary metastases. PET was included only during the last 3 years and not on a routine basis. Mediastinoscopy has not been performed routinely in this series unless the CT scan demonstrated mediastinal lymph node enlargement, PET suggested a malignant involvement of hilar or mediastinal nodes, or high- risk criteria of N2 were present. Bronchopulmonary, hilar, and mediastinal lymph nodes were systematically sampled. After surgery a final pathologic stage was determined based on operative findings.

Patients were classified into two groups: Group 1 (asymptomatic): patients who had no symptoms attributable to lung cancer at the time of imaging (patients whose cancer was detected by a medical checkup or under evaluation for other diseases), and Group B (symptomatic): patients with lung cancer-related symptoms. The charts of patients classified as having asymptomatic incidentally detected lung cancers were reviewed to check if the indications for imaging really were not based on any potentially cancer-related symptom.

Postoperative follow-up included office visits, quarterly chest X-rays, and yearly chest-CT. Operative or in-hospital mortality was defined as death occurring within 30 days after the operation or during hospitalization, respectively. All patients with postoperative or in-hospital mortality were included in this study.

### 2.1. Statistical Analysis

Statistical analysis was performed using SPSS 13.0 statistical software. The analysis of differences in categorical outcomes was determined using the Chi-squared test or Fisher's exact test. Probabilities of survival rates were estimated using the Kaplan-Meier method and ASX and SX patients were compared by using the log-rank test.

## 3. Results

Of 593 patients included in this study (68.3% male, median age 60.9, and range 23–86 years) 320 patients were asymptomatic (ASX) (53.9%). Two hundred and thirty (71.8% of the ASX patients) were diagnosed incidentally on chest X-ray and the remaining on CT scan. Amongst the patients with symptoms, the leading complaints that resulted in the indication for imaging were the appearance of new cough or the increase of a previously manifested clinical picture suggestive of pneumonia and haemoptysis (Table 1). Amongst the 320 ASX patients, the main reason for the imaging was a routine checkup (Table 2). Once the initial chest-X ray (71.8%) or CT scan (28.2%) showed an abnormal image, the usual workup for pulmonary nodules was started.

Patients in the ASX group were older than patients in SX group (median age 61.9 ± 9.9 versus 59.51 years/old ± 10.2, *P* = 0.007), without differences in sex (men 66 versus 73.5%, *P* = 0.084). They had a higher prevalence of previous malignancy (13.2 versus 4.8%, *P* = 0.002). The frequency of presentation as SPN (49.5 versus 19.4%, *P* < 0.001) or peripheral location (80.3 versus 63.7%, *P* < 0.001) was higher in this group, without differences in clinical suspicion of N2 (8.8 versus 12.9%, *P* = 0.146). Patients with incidentally detected lung cancer were more likely to have earlier-stage disease, smaller cancers (3.00 ± 2.2 versus 4.3 ± 2.9 cm, *P* = 0.0001). The incidence of adenocarcinoma (66.9% versus 53.8%; *P* = 0.025) was significantly higher in the ASX group. Clinical characteristics of both groups are shown in Table 3.

When the last ten years were analyzed, a higher prevalence of incidental detection compared to previous years was observed (51.7 versus 39.8%, *P* = 0.008).

**Figure 1:**
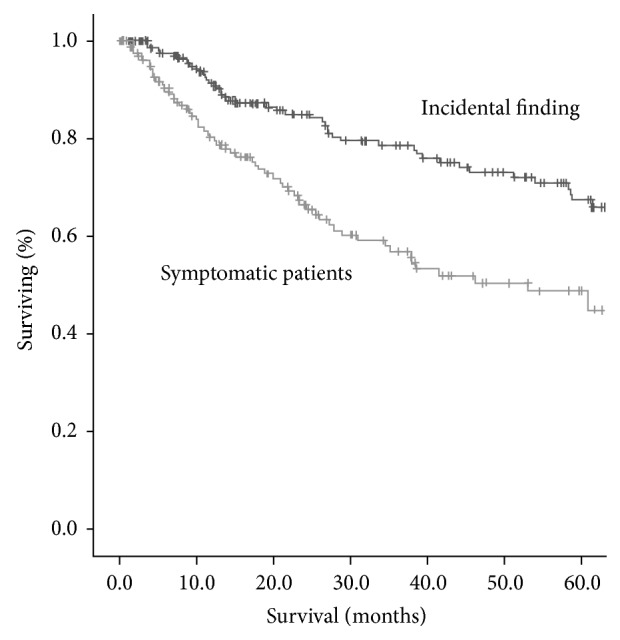
Overall survival curves of patients with lung cancer. The 5-year survival rates were 66.2% and 46.0% in ASX and symptomatic patients, respectively. Group I (ASX) had significantly more favorable prognoses (*P* = 0.001).

**Figure 2:**
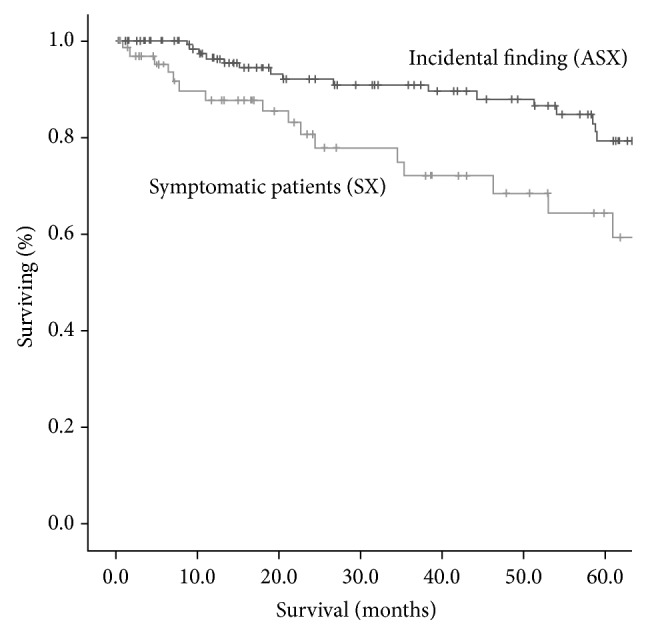
Overall survival curves of patients with pathologic stage I disease. Amongst the stage I patients, the 5-year survival rates were 81.2% in ASX patients and 58.6% in SX patients (*P* = 0.014).

**Figure 3:**
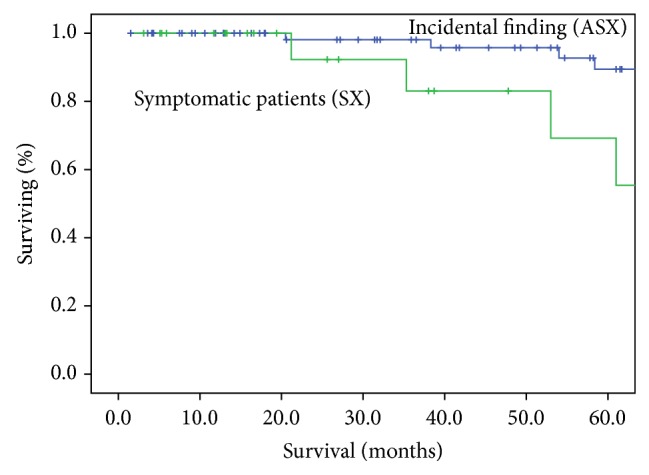
Overall survival curves of patients with pathologic stage IA disease. Amongst the stage IA patients, the 5-year survival rates were not different (89.2% in ASX patients and 71.8% in SX patients, *P* = 0.191).

The overall 5-year survival rates were higher for ASX patients: 66.2% and 46.0% for ASX and symptomatic patients, respectively (*P* = 0.001) (Figure 1). Amongst the stage I patients, the 5-year survival rates were 81.2% in ASX patients and 58.6% in SX patients (*P* = 0.014) (Figure 2). When only stage IA was considered, 5-year survival rates were not different (71.2 versus 84.1%, *P* = 0.191) (Figure 3). When analysis was restricted to T1a tumors there were no differences either in 5-year survival (94.7 versus 93.2, *P* = 0.489). Median survival times in pathological stages IIIB (41.6 m in ASX versus 22.0 m in SX patients, *P* = 0.065) and IV (13.7 versus 12.7 m, *P* = 0.964) were not significantly different.

## 4. Discussion

Our study shows that the incidental finding of non-small cell lung cancer occurred more frequently in smokers and in patients with a history of previous malignancy. There were a higher proportion of solitary nodules in stage I patients. The mortality of patients with NSCLC as an incidental diagnosis was lower, and this difference persisted into stage I.

More than a half of patients who underwent surgical resection of lung cancer at our institution had incidentally detected cancers and the most common indication for the initial imaging was a routine checkup. That proportion of ASX patients is higher than that reported by Raz et al. in San Francisco [7] but far lower than that published by Hanagiri et al. in Japan [8]. In the absence of a uniform policy appertaining to the role for screening in clinical practice, the indication of imaging asymptomatic patients relies on the preferences and beliefs of both patients and physicians. Different levels of awareness and access to healthcare may justify differences amongst different studied populations. Also, our ASX patients were slightly older (contrary to the study by Raz et al. [7]) and more frequently smokers which may mean a higher degree of awareness of their risk for lung cancer as the higher prevalence of previous malignancy may have been one of the reasons for routine radiological surveillance. However, it is noticeable that more than 70% of patients in our group (as in other series) were studied by chest-X-ray, a method that has proved to be ineffective and that is not recommended as a screening tool by any major medical organizations.

The proportion of early stages of lung cancer was higher amongst our patients with incidental findings. It has been also shown in the early report by Shimizu et al. that stage I cases accounted for 65.3% and stage III cases for 22% in their mass screened group, while, in the symptom group, there was only half that percentage of stage I cases (32.2%) [9]. Similarly, in the two more recently published series [7, 8] and in the Korean Lung Cancer Registry Study [10] asymptomatic patients had higher proportions of stages I-II. However, there were still 20% of our patients that did not have any symptom and had a stage III or IV lung cancer. Interestingly, in a retrospective review of coronial autopsies even when the median tumor size of previously undetected cancers was 3 cm, the range was 1–10 cm and there were several tumours over 5 cm and even some large endobronchial and hilar tumours undetected before death [11].

We found that survival time in symptomatic cases was worse than in incidentally detected lung cancer patients. The 5-year overall survival was lower for the whole group and for pathological stage I. That better outcome has been consistently demonstrated in all the previous reports; however, the causes for those differences are still unclear. The Korean registry [10] has shown that absence of symptoms at diagnosis significantly reduced the risk of death from NSCLC, regardless of age, gender, stage, smoking history, or whether treatment was performed. Similarly, Hanagiri and colleagues [8] showed that their patients with incidentally diagnosed NSCLC had significantly better prognoses than the symptomatic group even in stage II–IV disease cases. Even when they had a larger proportion of stage IV amongst asymptomatic patients than our series (6.7 versus 3.4%) they were still a small number of patients (*n* = 18) and exact figures of survival rates for those advanced patients were not provided. In our series, whilst pathological stage I patients had a better survival in ASX patients, median survival times in stages IIIB and IV were similar. It resembles the results of Raz et al. who found that their patients with completely resected incidental lung cancer had similar long-term survival rates as patients with symptomatic lung cancer, after adjusting for stage [7].

In the present study, stage IA disease was diagnosed less frequently in the symptomatic group, similar to what has been reported in other studies [7, 8, 10]. A study by Kashiwabara et al. [12] (published in 2002, before the publication of the 7th TNM edition) compared the outcomes in patients with one-year delayed detection of lung cancer on mass screening with chest-X-ray and in patients with no delay (patients with tumours which could versus could not be detected on past chest roentgenograms). They found that one-year delayed detection of lung cancer on mass screening did not affect outcome, but that, according to the maximum dimension of the tumours on the overlooked chest roentgenogram, the 5-year survival rates in patients with missed tumours were different and that survival in early stages (I-II) for missed tumours >20 mm was worse than that in patients with missed tumours <10 mm. We had previously shown that tumors over 15 mm are associated with shorter 5-year survival in all TNM stages [13] and several studies have reported tumor size may have an independent predictive value on survival in stage I patients [14, 15]. The impact of the tumor size was finally made evident by the analyses of the database of the IASLC and generated the reclassification of T1 in T1a and T1b and T2 in T2a and T2b [16]. When we analyzed separately the pathological stage IA cases, differences in survival in stage I between the two groups disappeared, suggesting that the size was the most important factor in determining survival.

Our study shows that patients with incidentally detected lung cancer had a better survival because they had smaller cancers and earlier-stage disease. The* clinical significance* of* these results* is difficult to interpret. The conclusions of clinical studies like this or any of the previously published studies should not be extrapolated to the potential value of mass screening. This sort of study design does not allow demonstrating if there is a survival benefit of treatments in asymptomatic patients or how large the proportion of invasive procedures for benign lesions is performed. One of the main concerns about any screening program is that a proportion of screen-detected cases will be “overdiagnosed” simply because of competing mortality [17], a hypothesis that cannot be excluded by a population study like this one. On the other hand, many of the patients in this and the other clinical series were not represented in the clinical trials about mass screening programs: 15% of our asymptomatic patients were never smokers and many of them were under 55 years old and would have not filled criteria for being included in a screening program. This study shows the importance of identifying risk at an individual level as many subjects different from the NLST participants may have a risk similar to or greater than the level of risk observed in NLST. Several studies have previously recognized that there is wide variation in lung cancer risk even amongst those who are smokers [18, 19] and we do not know yet how to identify other risk factors for lung cancer that could potentially justify extending screening to those individuals. Future research to develop clinically useful risk model might include molecular or genetic indicators of risk in order to answer these questions [20].

The National Lung Screening Trial (NLST) showed that, in heavy (30 pack-years or more) current or former (within 15 years) smokers between the ages of 55 and 75, three annual low-dose computed tomographic (LDCT) screens reduced lung cancer-specific mortality from 309 to 247 deaths per 100,000 person-years (relative risk of 0.8) [5]. But at the moment to make individual decisions (such as screening of certain nonsmokers) it is necessary to take into account the potential effectiveness of such measures. Whilst the number needed to screen (NNS) to prevent one death for the entire NLST population was calculated as 320, according to Bach and Gould for very low risk individuals (defined by the authors as a 40-year-old former smoker) the NNS was over 35,000 to prevent one lung cancer death [21]. In order to minimise the potential for harm when screening large populations for a condition that is very rare (derived not only from the costs [22] associated with screening but also from the impact on quality of life of the potential for invasive procedures for incidental findings) but at the same time not to miss other high-risk subjects out of the NSLT criteria, better risk models must be developed to have the greatest predictive accuracy for lung cancer risk.

This study has some limitations. Firstly, the classification of a cancer as incidentally detected is a potential bias, once the spontaneous patient consultation may not exclude the presence of some nonspecific symptom that prompted the patient to seek medical consultation. Secondly, the results from a single institution might not be generalizable.

It is remarkable that, in the study by Kashiwabara et al. [23] about patients that did not consult a physician after the discovery of a shadow in a radiological screening (almost 25% of the asymptomatic screened patients in their series), when asked about the reason why patients did not consult a doctor, two-thirds answered that it was because they did not have any respiratory symptoms. It shows that a screening program must assure that the health care system can provide all the necessary resources to treat the incidental findings and also the education to guarantee the availability of well-qualified primary care providers trained to encourage patients to follow diagnosis and treatment recommendations once a suspicion of lung cancer is raised from the imaging studies.

## 5. Conclusion

In summary, our study shows that lung cancer as an incidental finding is not uncommon even amongst nonsmokers and that the better survival of patients with asymptomatic NSCLC is related to the greater number of patients with earlier-stage disease. Future research is needed to prospectively identify those patients not represented in the NSLT who might benefit from LDCT screening.

## Figures and Tables

**Table 1:** Indications for imaging in symptomatic (SX) patients.

Leading symptom	*n*	%
Cough	113	41.39
Pneumonia	50	18.32
Hemoptysis	35	12.82
Dyspnea	21	7.69
Chest wall pain	13	4.76
Shoulder pain	6	2.20
Weight loss	9	3.30
Other symptoms	26	9.52

	273	100

**Table 2:** Indications for imaging in symptomatic (SX) patients.

Indication of imaging	*n*	%
Routine checkup	108	33.75
Preoperative CXR	42	13.12
Surveillance for cancer	42	13.12
Evaluation of chronic respiratory conditions	44	13.75
Evaluation of nonchest conditions or symptoms	66	20.62
Unknown	18	5.62

	320	100

**Table 3:** Characteristics of patients diagnosed after an incidental finding and patients with symptoms.

	Incidental finding *n* = 320		Symptomatic patients *n* = 273		*P*
Age (mean, SD)	61.93	9.8	59.51	10.1	0.007
Male (*n*, %)	212	66%	200	73%	0.054
Never smoker	50	15.6%	18	6.6%	0.003
Previous malignancy	41	13%	13	5%	0.002
Pathological staging (*n*, %)					
IA	121	37.81	36	13.19	0.0001
IB	71	22.19	52	19.05	
IIA	16	5.00	10	3.66	
IIB	40	12.50	50	18.32	
IIIA	46	14.38	86	31.50	
IIIB	15	4.69	24	8.79	
IV	11	3.44	15	5.49	
Squamous cell	51	16%	63	23.1%	0.031
Adenocarcinoma	214	66.9%	147	53.8%	0.025
Pneumonectomy	10	3.1%	28	11%	0.005
Tumor size >3 cm	149	46.5	200	73%	0.0001
Central tumor location	60	18.75	99	36.20%	0.001
Resection considered curative	288	90%	220	81%	0.007
Postoperative complication rate	57	17.8%	73	26.7%	0.022
ICU stay days (mean, SD)	1.73	3.209	1.44	4.388	0.38
Operative mortality	11	3.6%	17	6.2%	0.355
